# A cost of illness study evaluating the burden of Wolfram syndrome in the United Kingdom

**DOI:** 10.1186/s13023-019-1149-7

**Published:** 2019-07-31

**Authors:** Sana Eljamel, Wrik Ghosh, Sachin De Stone, Annabel Griffiths, Timothy Barrett, Richard Thompson

**Affiliations:** 1 Costello Medical, Cambridge, UK; 2 Institute of Cancer and Genomic Sciences, College of Medical and Dental Sciences, University of Birmingham, Birmingham, UK; 3 Findacure, Cambridge, CB1 2BL UK

**Keywords:** Wolfram syndrome, DIDMOAD, Cost of illness, Economic burden, rare disease

## Abstract

**Background:**

Wolfram syndrome is a rare genetic, progressive, neurodegenerative disorder characterised by childhood-onset diabetes mellitus, diabetes insipidus, optic atrophy and deafness. To date, the economic burden of Wolfram syndrome has not been well-studied or reported. The aim of this study was to evaluate the cost of illness (COI) of all people with Wolfram syndrome in the UK and to identify major determinants of cost from a service provider perspective (National Health Service, NHS).

**Methods:**

A prevalence-based approach was used to model the UK Wolfram syndrome specialist service. Model inputs were informed by a pragmatic literature review and UK reference costs, in conjunction with patient interviews and expert opinion. A deterministic sensitivity analysis (DSA) was run at 10% to identify major cost drivers.

**Results:**

The total COI of all people with Wolfram syndrome to the NHS was £1,055,899 per year, with an average annual cost per person with Wolfram syndrome of £16,498. Costs associated with diabetes mellitus care, late-stage diabetes mellitus complications and hearing impairment contributed most to the COI (18.9, 21.4 and 15.8% of the COI, respectively). The DSA identified costs associated with hearing impairment, diabetes mellitus care and end-stage renal disease (a diabetes mellitus complication) as major model drivers.

**Conclusions:**

The annual cost of Wolfram syndrome to the NHS was found to be substantial, with areas of potential cost savings identified, such as diabetes mellitus management. This model provides crucial information to facilitate economic evaluation of prospective therapies for this disease.

## Background

Wolfram syndrome is a rare, progressive, neurodegenerative disorder with an estimated UK prevalence of 1 in every 770,000 individuals [1]. This multisystem disease is characterised by 4 hallmark features: diabetes insipidus (DI), diabetes mellitus (DM), optic atrophy (OA) and deafness (D). These features give Wolfram syndrome its alternative name, DIDMOAD [2]. In addition to these hallmark DIDMOAD symptoms, patients often manifest a number of other symptoms, including psychiatric problems and neurologic abnormalities [2, 3].

Wolfram syndrome is a recessive genetic disorder, with two genetic types. People with Wolfram syndrome type 1 comprise almost all Wolfram syndrome cases. In these individuals, Wolfram syndrome is caused by mutations in the *WFS1* gene [1], which encodes the protein wolframin. Wolframin has many roles in the regulation of cellular processes such as cell death, protein folding and insulin production, and is a regulator of the unfolded protein response and endoplasmic reticulum calcium homeostasis [1]. A small number of Wolfram syndrome patients have Wolfram syndrome type 2, which is caused by mutations in the *CISD2* gene [1]. This gene encodes a protein found in the outer membrane of mitochondria [1]. Wolfram syndrome type 2 is prevalent in a distinct founder population in Jordan, who suffer similar symptoms to people with *WFS1* mutations, but with upper gastrointestinal ulcers and a bleeding disorder rather than DI and psychiatric disorders [2].

Individuals with Wolfram syndrome typically present with one or more of the main DIDMOAD symptoms, and diagnosis is confirmed by genetic testing of the *WFS1* gene; the *CISD2* mutation is not commonly screened for [1]. Typically, the earliest symptom is childhood-onset DM from age 6, followed by OA from age 11 and blindness by age 18–19 [2, 3]. The life expectancy of people with Wolfram syndrome was thought to be only 30 years; however, it is now known that some individuals live well into middle age [2, 4]. Data from the National Health Service (NHS) England highly specialised multidisciplinary service for Wolfram syndrome show that the median age of adults attending the specialised service is 37 years, with the oldest patient being 62 years. Death is commonly due to neurodegenerative atrophy of the brain stem [5, 6].

At present, there is no cure or disease-modifying treatment for Wolfram syndrome; the focus is only on the management of symptoms through existing treatments. NHS England highly specialised multidisciplinary services for Wolfram syndrome (paediatric and adults) are available at specialist centres in Birmingham, UK (Birmingham Women’s and Children’s Hospital and Queen Elizabeth Hospital, respectively). These specialist services allow monitoring of disease progression, provision of treatments, advice regarding symptom management and participation in registry studies and clinical trials.

To date, the economic burden of Wolfram syndrome has not been well-studied or reported. As yet, there are no published cost of illness (COI) studies investigating Wolfram syndrome. Such studies are an important first step in understanding the current resource use and to inform the UK’s NHS resource allocation. COI studies can be a valuable addition to the evidence base in rare and neglected genetic conditions, and help to provide support for the research of new treatments.

The purpose of this study was to estimate the COI of Wolfram syndrome to the NHS and Personal Social Services, based on the treatment pathway followed by patients receiving NHS care, typically at the specialist centres for children and adults in Birmingham. The key cost and clinical inputs of the model are highlighted and the factors leading to the largest contribution to annual costs are discussed.

## Methods

A COI model was developed in Microsoft Excel® 2016 (Microsoft, Redmond, Washington) to calculate the annual direct costs to the NHS and Personal Social Services incurred by all individuals diagnosed with Wolfram syndrome in the UK at the time of the model development. A prevalence-based approach was used to estimate the annual cost associated with the diagnosis of Wolfram syndrome, the treatment of Wolfram syndrome symptoms and the running of the Wolfram syndrome specialist services at the Birmingham Children’s Hospital and Queen Elizabeth Hospital.

**Fig. 1:**
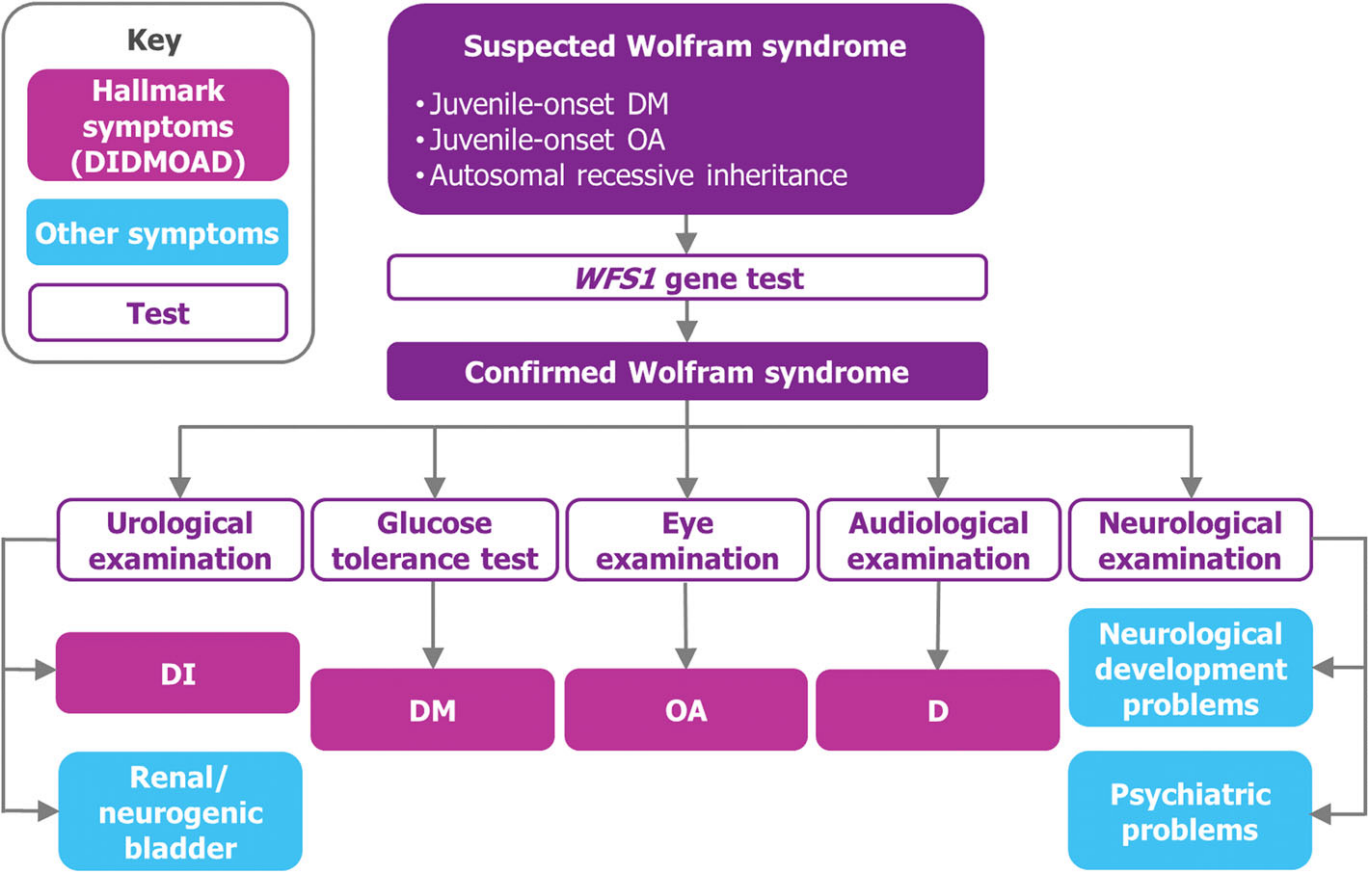
Costs associated with different symptom groups. *D* deafness, *DI* diabetes insipidus, *DIDMOAD* diabetes insipidus, diabetes mellitus, optic atrophy and deafness (alternative name for Wolfram syndrome), *DM* diabetes mellitus, *OA* optic atrophy

The treatment pathway was divided into diagnosis and referral processes, running of specialist services and treatment of symptom groups (Fig. 1). The typical ‘per person with Wolfram syndrome cost’ for each of these resources was calculated by multiplying the expected resource use with the unit cost. Resource use was defined as the use of healthcare staff time, facilities, or consumables such as medicines. The unit cost refers to the cost per ‘unit’ of resource, e.g. the cost per consultation, cost per hour of nursing time, cost per blood test or cost per box of medicine. The ‘per person with Wolfram syndrome cost’ was then multiplied by the number of individuals expected to require the resource in question, with the expected service utilisation based on the age group distribution of affected individuals and the symptoms expected in each age group. The number of patients expected to have a particular symptom was estimated by excluding any patients younger than the median age of symptom onset. The total COI was calculated by summing costs for each service, i.e. diagnosis and referral, specialist services and treatment of symptom groups.

### Model inputs

**Fig. 2:**
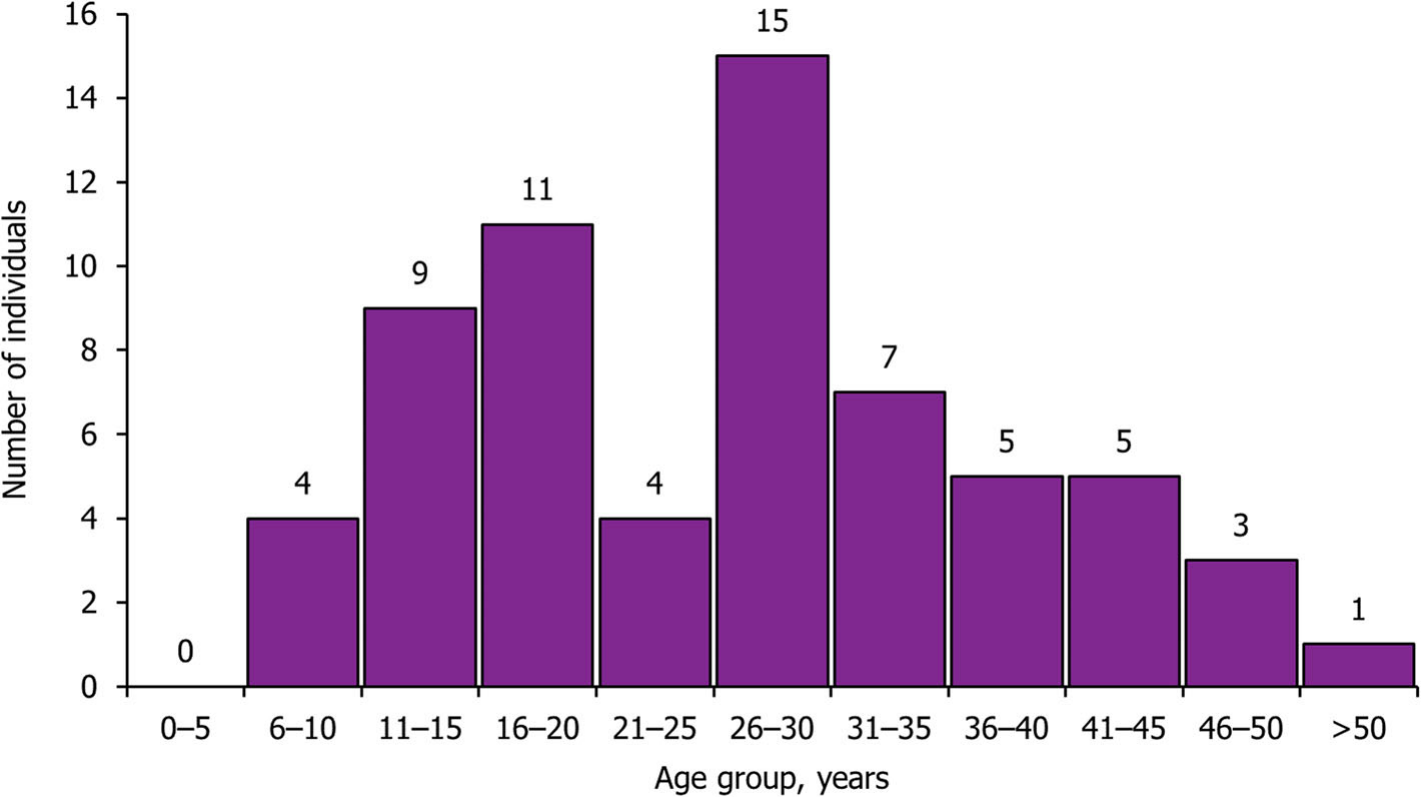
Age distribution of individuals with Wolfram syndrome. Age distribution estimate based on routine data from NHS Wolfram Syndrome Services at Birmingham Children’s Hospital and Queen Elizabeth Hospital

Clinical inputs, such as the number of children and adults diagnosed with Wolfram syndrome and the age distribution of Wolfram syndrome patients, were sourced from published literature, routine data from Wolfram specialist centres and clinical experts (Table 1 and Fig. 2) [2–4, 7–30].

**Table 1:** Clinical inputs of the model

Input	Value	Reference
DM care		
Median age of DM onset, years	6	Barrett et al. 1995 [2]
Proportion of patients managed by multiple daily injections	0.81	Diabetes UK, 2012 [7]
Proportion of patients managed by continuous subcutaneous insulin infusions	0.19	Diabetes UK, 2012 [7]
Prevalence of ketoacidosis	0.07	Rohayem et al. 2011 [8]
Frequency of severe hypoglycaemia	0.37	Rohayem et al. 2011 [8]
Proportion of patients with symptoms suggestive of coeliac disease	0.25	Tranebjaerg et al. 2013 [9]
DM complications		
Incidence of myocardial infarction	0.32	Pratoomsoot et la. 2009 [10]
Incidence of angina	0.08	Pratoomsoot et la. 2009 [10]
Incidence of congestive heart failure	0.24	Pratoomsoot et la. 2009 [10]
Incidence of fatal stroke	0.04	Pratoomsoot et la. 2009 [10]
Incidence of non-fatal stroke	0.08	Pratoomsoot et la. 2009 [10]
Incidence of peripheral vascular disease	0.16	Pratoomsoot et la. 2009 [10]
Incidence of end-stage renal disease	0.31	Pratoomsoot et la. 2009 [10]
Incidence of proliferative diabetic retinopathy	0.33	Pratoomsoot et la. 2009 [10]
Incidence of diabetic-induced cataract development	0.12	Pratoomsoot et la. 2009 [10]
Incidence of neuropathy	0.90	Pratoomsoot et la. 2009 [10]
Incidence of ulcer	0.47	Pratoomsoot et la. 2009 [10]
Incidence of major hypoglycaemic event (severe hypoglycaemia)	0.14	Pratoomsoot et la. 2009 [10]
DI and other endocrine disorders care		
Median age of DI onset, years	16	Barrett et al. 1995 [2]
Proportion of patients with DI	0.72	Barrett et al. 1995 [2]
Proportion of patients with hypo- or hypergonadotropic hypogonadism	0.34	Rohayem et al. 2011 [8]
Proportion of patient with hypogonadism, male	0.75	Tranebjaerg et al. 2013 [9]
Proportion of patients with hypogonadism, female	0.25	Tranebjaerg et al. 2013 [9]
Visual impairment care		
Median age of OA onset, years	10.0	Barrett et al. 1995 [2]
Median time to blindness, years	8.00	Barrett et al. 1995 [2]
Proportion of patients developing cataracts	0.05	Chaussenot et al. 2011 [3]
Hearing impairment care		
Median age of hearing impairment onset, years	13	Barrett et al. 1995 [2]
Median time from hearing impairment to severe hearing loss, years	12.0	Expert opinion (Professor Timothy Barrett), 2016
Proportion of patients with SNHL	0.66	Barrett et al. 1995 [2]
Proportion of patients with SNHL requiring cochlear implants	0.17	Karzon et al. 2013 [11]
Renal/neurogenic bladder care		
Median age of neurogenic bladder onset, years	15	Expert opinion (Professor Timothy Barrett), 2019
Proportion of patients with neurogenic bladder	0.55	Barrett et al. 1995 [2]
Proportion of patients with neurogenic bladder managed on clean intermittent self-catheterisation	0.31	Assumes all patients > 10 years old without visual impairment are able to self-catheterise
Proportion of patients with neurogenic bladder managed on an indwelling catheter	0.69	Assumes all patients > 10 years old without visual impairment are able to self-catheterise
Proportion developing symptomatic UTI, out of patients with neurogenic bladder	0.68	NCGC Infection prevention and control, 2012 [12]
Proportion developing urethral complication, out of patients with neurogenic bladder	0.02	NCGC Infection prevention and control, 2012 [12]
Proportion with first-line antibiotic resistant UTI, out of patients with neurogenic bladder	0.09	NCGC Infection prevention and control, 2012 [12]
Proportion with multidrug resistant UTI, out of patients with neurogenic bladder	0.07	NCGC Infection prevention and control, 2012 [12]
Proportion developing bacteraemia secondary to UTI, out of patients with neurogenic bladder	0.04	NCGC Infection prevention and control, 2012 [12]
Neurological care		
Median age of neurological symptom onset, years	15	Chaussenot et al. 2011 [3]
Proportion of patients with truncal/gait ataxia	0.33	Barrett et al. 1995 [2]
Proportion of patients with areflexia of the lower limb	0.20	Barrett et al. 1995 [2]
Proportion of patients with severe startle myoclonus requiring wheelchair	0.04	Barrett et al. 1995 [2]
Proportion of patients with central apnoeas	0.11	Barrett et al. 1995 [2]
Proportion of patients with cerebellar dysarthria	0.11	Barrett et al. 1995 [2]
Proportion of patients with autonomic neuropathy	0.09	Barrett et al. 1995 [2]
Proportion of patients with hemiparesis	0.09	Barrett et al. 1995 [2]
Psychological care		
Median age at psychological/psychiatric disorder onset, years	14	Rohayem et al. 2011 [8]
Proportion of patients with psychological/psychiatric disorders	0.60	Swift et al. 1990 [13]
Proportion of patients with severe psychiatric disorders requiring hospitalisation	0.29	Swift et al. 1990 [13]
Proportion of patients with learning difficulties	0.24	Rohayem et al. 2011 [8]

Incidence estimates used in the model were calculated by adjusting cumulative incidence rates to incidence per patient per year. *DI* diabetes insipidus, *DM* diabetes mellitus, *NCGC* National Clinical Guideline Centre, *OA* optic atrophy, *SNHL* sensorineural hearing loss, *UTI* urinary tract infection

Based on routine records from the Birmingham Children’s Hospital, there were at least 64 individuals diagnosed with Wolfram syndrome at the time of the COI model development. Nine further paediatric patients are expected to be newly diagnosed and seen at the specialist centre at Birmingham Children’s Hospital annually. Each year, 15 paediatric patients and 14 adult patients are seen as follow-up patients at specialist clinics. In addition, 7 previously diagnosed paediatric patients are expected to transition to adult services at the specialist centre at Queen Elizabeth Hospital each year. These numbers are averages based on audit data for patients seen at Wolfram syndrome specialist clinics from March 2014 to November 2015. Individuals in the UK are typically diagnosed with Wolfram syndrome type 1 and thus individuals with Wolfram syndrome type 2 were not considered in this model.

The services and resource use required for diagnosis, annual review and management of symptoms were obtained from published literature, as well as clinical experts from the specialist Wolfram centre at Birmingham Children’s Hospital (Table 2). Unit costs were derived from NHS Reference Costs (2015-16) [19], British National Formulary (BNF, 2016) [14], Personal Social Services Research Unit 2016 [25] and the literature. A small number of unit cost inputs were estimated from expert opinion (Professor Timothy Barrett) in the absence of published inputs. All clinical inputs were verified by clinical experts at the specialist Wolfram centre at Birmingham Children’s Hospital. Feedback from these experts was obtained via face-to-face meetings and questionnaire completion. Where applicable, all costs were inflated to correspond to 2016 costs. Wolfram syndrome and its associated treatments were assumed to have no effect on mortality: a mortality rate of 0% was applied to all patients and procedures.

**Table 2:** Cost and resource use inputs of the model

Input	Unit Cost	Resource Use	Reference
Diagnosis			
Outpatient appointment at local tertiary hospital	£330.14 [19]	1 appointment for each new patient	NHS Reference Costs 2015–2016 [19] NHS UK Genetic Testing Network, 2016 [27]
Genetic testing, *WFS1* sequencing	£550.00 [27]		NHS Reference Costs 2015–2016 [19] NHS UK Genetic Testing Network, 2016 [27]
Referral to specialised service (paediatric)			
Blood tests (serum osmolality, glucose, HbA1c)	£1.18 [19]		NHS Reference Costs 2015–2016 [19]
Urine osmolality test	£1.18 [19]		NHS Reference Costs 2015–2016 [19]
Genetic counselling	£481.89 [19]		NHS Reference Costs 2015–2016 [19]
MRI scan, brain	£142.99 [19]		NHS Reference Costs 2015–2016 [19]
Orthoptics assessment	£66.05 [19]		NHS Reference Costs 2015–2016 [19]
Retinal photography	£174.16 [19]		NHS Reference Costs 2015–2016 [19]
Ophthalmologist review	£118.59 [19]		NHS Reference Costs 2015–2016 [19]
Psychology assessment	£249.26 [19]	1 appointment for each new patient	NHS Reference Costs 2015–2016 [19]
Audiological examination	£151.69 [19]		NHS Reference Costs 2015–2016 [19]
Auditory brain response (ABR) testing	£29.19 [19]		NHS Reference Costs 2015–2016 [19]
ENT review	£122.78 [19]		NHS Reference Costs 2015–2016 [19]
Consultant review	£330.14 [19]		NHS Reference Costs 2015–2016 [19]
Diabetic specialist review	£391.00 [19]		NHS Reference Costs 2015–2016 [19]
Urological examination	£102.41 [19]		NHS Reference Costs 2015–2016 [19]
Urology review	£124.55 [19]		NHS Reference Costs 2015–2016 [19]
Specialised service (paediatric), annual review			
Blood tests (serum osmolality, glucose, HbA1c)	£1.18 [19]	1 specialist paediatric review per patient per year [16]	NHS Reference Costs 2015–2016 [19] NHS Wolfram Syndrome Service (Birmingham Children’s Hospital), 2016 [16]
Urine osmolality test	£1.18 [19]		As above
Genetic counselling	£404.29 [19]		As above
MRI scan, brain	£142.99 [19]		As above
Orthoptics assessment	£65.21 [19]		As above
Retinal photography	£174.16 [19]		As above
Ophthalmologist review	£115.31 [19]		As above
Psychology assessment	£195.59 [19]		As above
Audiological examination	£151.69 [19]		As above
Auditory brain response (ABR) testing	£29.19 [19]		As above
ENT review	£102.65 [19]		As above
Consultant review	£228.68 [19]		As above
Diabetic specialist review	£252.57 [19]		As above
Urological examination	£102.41 [19]		As above
Urology review	£114.81 [19]		As above
Specialised service (paediatric), additional costs			
Wellchild family liaison officer, per hour	£59.00 [25]	33 h per clinic	PSSRU, 2016 [25] NHS Wolfram Syndrome Service (Birmingham Children’s Hospital), 2016 [16] Expert opinion (Professor Timothy Barrett), 2016 NHS Reference Costs 2015–2016 [19]
Nurse specialist, per hour	£44.00 [25]	15 h per clinic [16]	As above
Research nurse, per hour	£44.00 [25]		As above
Clinic co-ordinator, per hour	£29.00 [25]	477 h per clinic	As above
MDT meeting, per patient	£107.35 [19]	2 MDT meetings per clinic 6 patients discussed at MDT meetings [16]	As above
Consultant, per hour	£104.00 [25]	6 h per clinic	As above
Number of paediatric specialist clinics, per year	–	4.00 [16]	As above
Transition services			
Cost of transition services to adulthood, per year	£979.00 [25]	1 per new patient seen at adult specialist centre	PSSRU, 2016 [25]
Specialised service (adult), new patients			
Blood tests (serum osmolality, glucose, HbA1c)	£1.18 [19]	1 per new patient seen at adult specialist centre	NHS Reference Costs 2015–2016 [19]
Urine osmolality test	£1.18 [19]		NHS Reference Costs 2015–2016 [19]
Genetic counselling	£481.89 [19]		NHS Reference Costs 2015–2016 [19]
Psychology assessment	£249.26 [19]		NHS Reference Costs 2015–2016 [19]
Consultant review	£195.87 [19]		NHS Reference Costs 2015–2016 [19]
Neurological review	£216.58 [19]		NHS Reference Costs 2015–2016 [19]
Specialised service (adult), follow-up review			
Blood tests (serum osmolality, glucose, HbA1c)	£1.18 [19]	0.75 follow-up appointments per patient per year [17]	NHS Reference Costs 2015–2016 [19] NHS Wolfram Syndrome Service (Queen Elizabeth Hospital), 2016 [17]
Urine osmolality test	£1.18 [19]		As above
Genetic counselling	£404.29 [19]		As above
Psychology assessment	£195.59 [19]		As above
Consultant review	£150.38 [19]		As above
Neurological review	£160.76 [19]		As above
DM care			
Paediatric outpatient diabetes care, per year	£2925.00 [20]	N/A (costs given are per patient with DM per year)	NHS National Tariff 2016–2017 [20] Cummins et al. 2010 [15] Patient experience (Wolfram Focus Group), 2016 [30] NHS Reference Costs 2015–2016 [19] Expert opinion (Professor Timothy Barrett), 2016 NHS Vale of York CCG, 2015 [21] NHS Southern Derbyshire CCG, 2016, UK [24]
Insulin treatment, multiple daily injections, average annual cost of consumables	£984.10 [15]		As above
Insulin treatment, continuous subcutaneous insulin infusions, average annual cost of consumables	£2863.85 [15]		As above
Endocrinologist outpatient appointment for diabetes management, adult services	£150.38 [19]	2 appointments per year [30]	As above
Diabetic specialist nurse appointment, adult services	£70.59 [19]	2 appointments per year	As above
Diabetic specialist nurse telephone appointment, adult services	£29.90 [19]	2 appointments per year	As above
Diabetic education service appointment	£44.33 [19]	1 appointment per year	As above
Coeliac serology, IgA tTG test	£23.75 [21]	1 per patient with symptoms suggestive of coeliac disease per year	As above
Coeliac serology, EMA test	£11.62 [21]		As above
Duodenal biopsy	£868.79 [19]	1 per patient with coeliac disease per year	As above
Dietician, community health service	£81.32 [19]	3 visits for coeliac disease per year [24]	As above
Ketoacidosis, inpatient care	£600.03 [19]	1 per patient with ketoacidosis per year	As above
Severe hypoglycaemic events, inpatient care	£413.39 [19]	1 per patient with hypoglycaemic event per year	As above
DM complications			
Myocardial infarction, year of event	£6234.64 [26]	N/A	Roze et al. 2005 [26]
Myocardial infarction, each subsequent year	£1027.06 [26]		Roze et al. 2005 [26]
Angina, year of onset	£3234.06 [26]		Roze et al. 2005 [26]
Angina, each subsequent year	£1067.37 [26]		Roze et al. 2005 [26]
Congestive heart failure, year of onset	£3606.53 [26]		Roze et al. 2005 [26]
Congestive heart failure, each subsequent year	£1229.97 [26]		Roze et al. 2005 [26]
Stroke, fatal	£4810.09 [26]		Roze et al. 2005 [26]
Stroke, year of event	£3813.61 [26]		Roze et al. 2005 [26]
Stroke, each subsequent year	£7225.56 [26]		Roze et al. 2005 [26]
Peripheral vascular disease, onset	£3321.62 [26]		Roze et al. 2005 [26]
Haemodialysis	£35,352.31 [26]		Roze et al. 2005 [26]
Peritoneal dialysis	£26,543.77 [26]		Roze et al. 2005 [26]
Kidney transplant, first year	£27,795.98 [26]		Roze et al. 2005 [26]
Kidney transplant, subsequent year	£9151.83 [26]		Roze et al. 2005 [26]
Retinal photocoagulation	£958.96 [26]		Roze et al. 2005 [26]
Cataract extraction	£2208.39 [26]		Roze et al. 2005 [26]
Cataract annual follow-up	£148.71 [26]		Roze et al. 2005 [26]
Neuropathy, onset	£1352.27 [26]		Roze et al. 2005 [26]
Uninfected ulcer	£1778.94 [26]		Roze et al. 2005 [26]
Infected ulcer	£1823.42 [26]		Roze et al. 2005 [26]
Major hypoglycaemic event	£307.15 [26]		Roze et al. 2005 [26]
DI and other endocrine disorders care			
Blood tests (serum osmolality, glucose, HbA1c)	£1.18 [19]	1 appointment for each new patient	NHS Reference Costs 2015–2016 [19] BNF, March 2016 [14] BNF, May 2016 [14] PSSRU, 2016 [25]
Desmopressin acetate, nasal spray, 10 micrograms/metered spray, 60 metered sprays	£8.76 [14]	2 metered sprays per day [14]	As above
Testosterone enanthate, 250 mg/1 mL solution for injection ampoules, 3 ampoules	£72.50 [14]	1 ampoule per month per male paediatric patient with hypogonadism	As above
Testosterone undecanoate, 250 mg/1 mL solution for injection, 1000 mg/4 mL, 1 vial	£80.00 [14]	1 g per 12 weeks per male adult patient with hypogonadism	As above
GP nurse attendance for testosterone administration, per visit	£9.30 [25]	1 per injection	As above
Estradiol valerate 1 mg, 28 tablets	£3.53 [14]	1 per 3 months per female patient with hypogonadism	As above
Desogestrel 75 microgram, 84 tablets	£5.21 [14]	1 per 3 months per female patient with hypogonadism	As above
Visual impairment care			
Optometry assessment	£95.84 [19]	2 per year per patient both before and after symptom onset [30]	NHS Reference Costs 2015–2016 [19] Patient experience (Wolfram Focus Group), 2016 [30] Federation of (Ophthalmic and Dispensing) Opticians, 2016 [18] Warren 1995 [28]
Retinal photography	£174.16 [19]		As above
Ophthalmologist review at local centre	£115.31 [19]		As above
Glasses, single vision lenses, ≤6 dioptres SPH	£39.10 [18]	1 averaged cost per patient per year	As above
Glasses, single vision lenses, > 6–≤10 dioptres SPH	£59.30 [18]		As above
Glasses, single vision lenses, > 10–≤14 dioptres SPH	£86.90 [18]		As above
Glasses, single vision lenses, > 14 dioptres SPH	£196.00 [18]		As above
Cataract surgery	£875.11 [19]	1 per patient requiring cataract surgery	As above
Low vision services assessment	£78.31 [19]	1 per patient requiring visual impairment care	As above
Occupational therapist visit	£131.02 [19]	6 visits per year per patient requiring visual impairment care [28]	As above
Hearing impairment care			
Audiological examination	£151.69 [19]	1 per year per patient both before and after symptom onset [30]	NHS Reference Costs 2015–2016 [19] Patient experience (Wolfram Focus Group), 2016 [30]
Auditory brain response (ABR) testing	£29.19 [19]		As above
ENT annual review	£102.65 [19]		As above
Audiology hearing aid fitting	£89.58 [19]	1 per patient requiring hearing aid	As above
Audiology hearing aid follow-up	£96.90 [19]		As above
Audiology annual review	£358.31 [19]	1 per patient with SNHL per year	As above
Cochlear implants, bilateral	£31,481.77 [19]	1 per patient requiring cochlear implant	As above
ENT follow-up post-implant for external cochlear implant fitting	£89.14 [19]		As above
Audiology follow-up post-implant, maintenance and programming	£291.73 [19]		As above
ENT annual review for severe hearing loss	£89.14 [19]		As above
Renal/neurogenic bladder care			
Urodynamic studies, paediatrics	£102.41 [19]	1 review per patient per year prior to symptom onset [30] 2 reviews per patient per year after symptom onset [29]	NHS Reference Costs 2015–2016 [19] Patient experience (Wolfram Focus Group), 2016 [30] Wolfram Syndrome Guideline Development Group, 2014, Europe [29] NCGC Infection prevention and control, 2012 [12] NCGC Urinary incontinence in neurological disease, 2012 [12] NHS Drug Tariff, May 2016 [22]
Urodynamic studies, adult services	£134.75 [19]		As above
Urology review, paediatrics	£114.81 [19]		As above
Urology review, adult services	£99.79 [19]		As above
Urine dipstick	£1.18 [19]		As above
Intermittent self-catheterisation, mean annual cost	£2771.58 [12]	Annual cost for patients managed by clean intermittent self-catheterisation [12]	As above
Indwelling catheter	£5.22 [12]	7 indwelling catheter changes per patient per year with indwelling catheter [22]	As above
Catheter leg bag	£2.39 [22]	60 drainage bag changes per patient per year with neurogenic bladder [22]	As above
Catheter drainage bag	£1.10 [22]		As above
Annual cost of symptomatic UTI	£40.06 [12]	Annual cost for patients with different forms of UTI and urethral complication	As above
Annual cost of first-line antibiotic resistant UTI	£60.42 [12]		As above
Annual cost of multidrug resistant UTI	£2232.50 [12]		As above
Annual cost of bacteraemia secondary to UTI	£3535.95 [12]		As above
Annual cost of urethral complication	£1402.07 [12]		As above
Mental health care initial assessment	£257.59 [19]	1 appointment per patient after symptom onset [30]	As above
Community mental health care, adult	£120.61 [19]	1 appointment per patient per year after symptom onset [30]	As above
Inpatient treatment of psychiatric disorders, per bed day	£390.20 [19]	15 days review period per patient [30]	As above
Rehabilitation of psychiatric disorders	£377.77 [19]	1 appointment per patient per year after symptom onset [30]	As above
Neurological care			
Neurological review, paediatric	£343.79 [19]	1 review per patients per year prior to symptom onset [29] 2 reviews per patient per year after symptom onset [4]	NHS Reference Costs 2015–2016 [19] Wolfram Syndrome Guideline Development Group, 2014, Europe [29] Urano et al. 2016 [4] Expert opinion (Professor Timothy Barrett), 2016 NHS Bolton Foundation Trust, 2015 [23]
Neurological review, adult services	£160.76 [19]		As above
Specialist nurse, paediatric, community health services	£137.36 [19]	2 appointments per patient per year after symptom onset	As above
Specialist nurse, telephone appointment, paediatric, community health services	£43.23 [19]	2 appointments per patient per year after symptom onset	As above
Specialist nurse, adult services, community health services	£77.24 [19]	1 appointment per patient per year after symptom onset	As above
Specialist nurse, adult services, telephone appointment, community health services	£33.08 [19]		As above
Physiotherapy, first appointment	£56.60 [19]		As above
Physiotherapy, follow-up	£45.86 [19]		As above
Speech and language therapist, first appointment	£87.04 [19]		As above
Speech and language therapist, follow-up	£99.32 [19]		As above
Occupational therapist, first appointment	£142.11 [19]		As above
Occupational therapist, follow-up	£58.25 [19]		As above
Clinical physiology, first appointment	£76.39 [19]		As above
Clinical physiology, follow-up	£51.58 [19]		As above
Gastroenterologist, first appointment	£164.54 [19]		As above
Gastroenterologist, follow-up	£132.50 [19]		As above
Podiatry, community health services	£39.88 [19]	1 appointment per patient per year after symptom onset and during late-stage symptoms	As above
Rehabilitation services	£94.26 [19]	1 appointment per patient per year after symptom onset	As above
MRI scan, brain and spine	£133.64 [19]	1 appointment per patient per year after symptom onset	As above
Nerve conduction studies, paediatric	£281.58 [19]		As above
Nerve conduction studies, adult services	£206.71 [19]		As above
Sleep studies	£501.71 [19]		As above
Bronchoscopy, paediatric	£1589.23 [19]		As above
Bronchoscopy, adult services	£632.10 [19]		As above
Tracheostomy insertion	£3489.92 [19]		As above
Orthopaedics, paediatrics, first appointment	£135.97 [19]	1 appointment per patient per year after symptom onset	As above
Orthopaedics, adult services, first appointment	£135.74 [19]	1 appointment per patient per year after symptom onset	As above
Orthopaedics, adult services, follow-up	£109.51 [19]		As above
Walking aids	£7.60 [23]	1 per patient requiring wheelchair or walking aids	As above
Wheelchair, assessment, paediatric	£309.04 [19]		As above
Wheelchair, equipment, paediatric	£485.57 [19]		As above
Wheelchair, maintenance, paediatric	£66.47 [19]		As above
Wheelchair, review, paediatric	£249.11 [19]		As above
Wheelchair, assessment	£247.21 [19]		As above
Wheelchair, equipment	£167.64 [19]		As above
Wheelchair, maintenance	£60.03 [19]		As above
Wheelchair, review	£151.66 [19]		As above
Psychological/psychiatric care			
Community mental health care, paediatric	£242.38 [19]	12 appointments per patient with symptoms per year [30]	NHS Reference Costs 2015–2016 [19] Patient experience (Wolfram Focus Group), 2016 [30]
Mental health care initial assessment	£257.59 [19]	1 appointment per patient after symptom onset [30]	As above
Community mental health care, adult	£120.61 [19]	1 appointment per patient per year after symptom onset [30]	As above
Inpatient treatment of psychiatric disorders, per bed day	£390.20 [19]	15 days review period per patient [30]	As above
Rehabilitation of psychiatric disorders	£377.77 [19]	1 appointment per patient per year after symptom onset [30]	As above

*ABR* auditory brain response, *BNF* British National Formulary, *CCG* Clinical Commissioning Group, *DI* diabetes insipidus, *DM* diabetes mellitus, *EMA* endomysial antibody, *ENT* ear, nose and throat, *GP* general practitioner, *HbA1c* haemoglobin A1c, *IgA* immunoglobulin A, *MDT* multi-disciplinary team, *MRI* magnetic resonance imaging, *NCGC* National Clinical Guideline Centre, *NHS* National Health Service, *PSSRU* Personal Social Services Research Unit, *SALT* Speech and Language Therapist, *SPH* sphere, *tTG* tissue transglutaminase, *UTI* urinary tract infection, *WFS1* Wolfram syndrome type 1 (gene)

### Deterministic sensitivity analysis

A univariate DSA was carried out to assess the sensitivity of the model results to variations in each of the model inputs. The values of the parameters were varied, one at a time, by ±10% to assess the impact of these changes on the total annual COI estimated by the model. The parameters were ranked in order of largest to smallest impact on costs when varied. These parameters represent the ‘cost drivers’ of the model.

## Results

### Cost of Wolfram syndrome

**Fig. 3:**
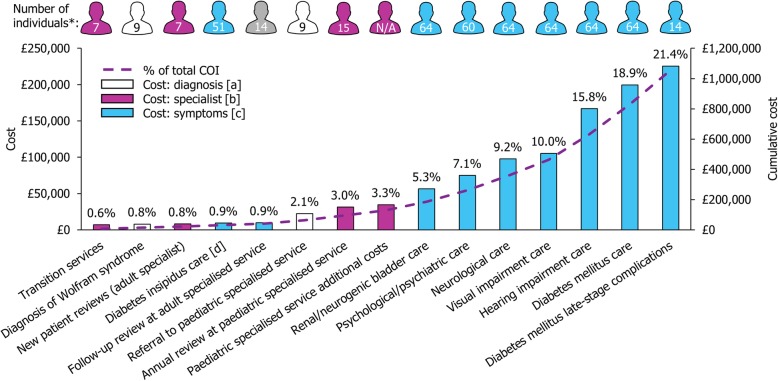
Resource use costs associated with individuals with Wolfram syndrome. [a] Diagnosis and referral to specialist services; [b] Specialist services; [c] Symptom management; [d] Includes other endocrine disorders care

The estimated annual costs for people with Wolfram syndrome are shown in Fig. 3. The total annual COI to the NHS was £1,055,899 per year, and the average cost of Wolfram syndrome to the NHS per person with Wolfram syndrome per year was £16,498. The costs of treatment of symptoms (£935,350) represented 88.6% of the total costs, with the greatest annual costs being associated with DM care (£199,640; 18.9%) and the treatment of late-stage DM complications (£225,459; 21.4%).

Costs considered for DM care included the costs of insulin treatment, consultations with endocrinologists, diabetes specialist nurse services, diabetes education, gastrointestinal examinations, dietician appointments and inpatient care for ketoacidosis and severe hypoglycaemic events. Twelve late-stage complications, such as myocardial infarction, end-stage renal disease, neuropathy and major hypoglycaemic event, were included in the model [10]. Hearing impairment care was also a key contributor to costs: the annual costs associated with hearing impairment care made up 15.8% of the total annual costs of all Wolfram syndrome patients (Fig. 3). As a percentage of the total annual costs of Wolfram syndrome patients, the remaining annual costs related to treatment of symptom groups were: 10.0%, visual impairment care; 9.2%, neurological care; 7.1%, psychological/psychiatric care; 5.3%, renal/neurogenic bladder care; and 0.9%, DI and other endocrine disorders care.

The costs of diagnosis and referral to specialist centres (2.9% of total costs), and the costs of providing specialist services (8.5% of total costs; this includes the cost of annual review services, the cost of running specialist centres and the cost of transition services for children when transferring to adulthood) only contributed to a minor proportion of the total costs.

### Deterministic sensitivity analysis

**Fig. 4:**
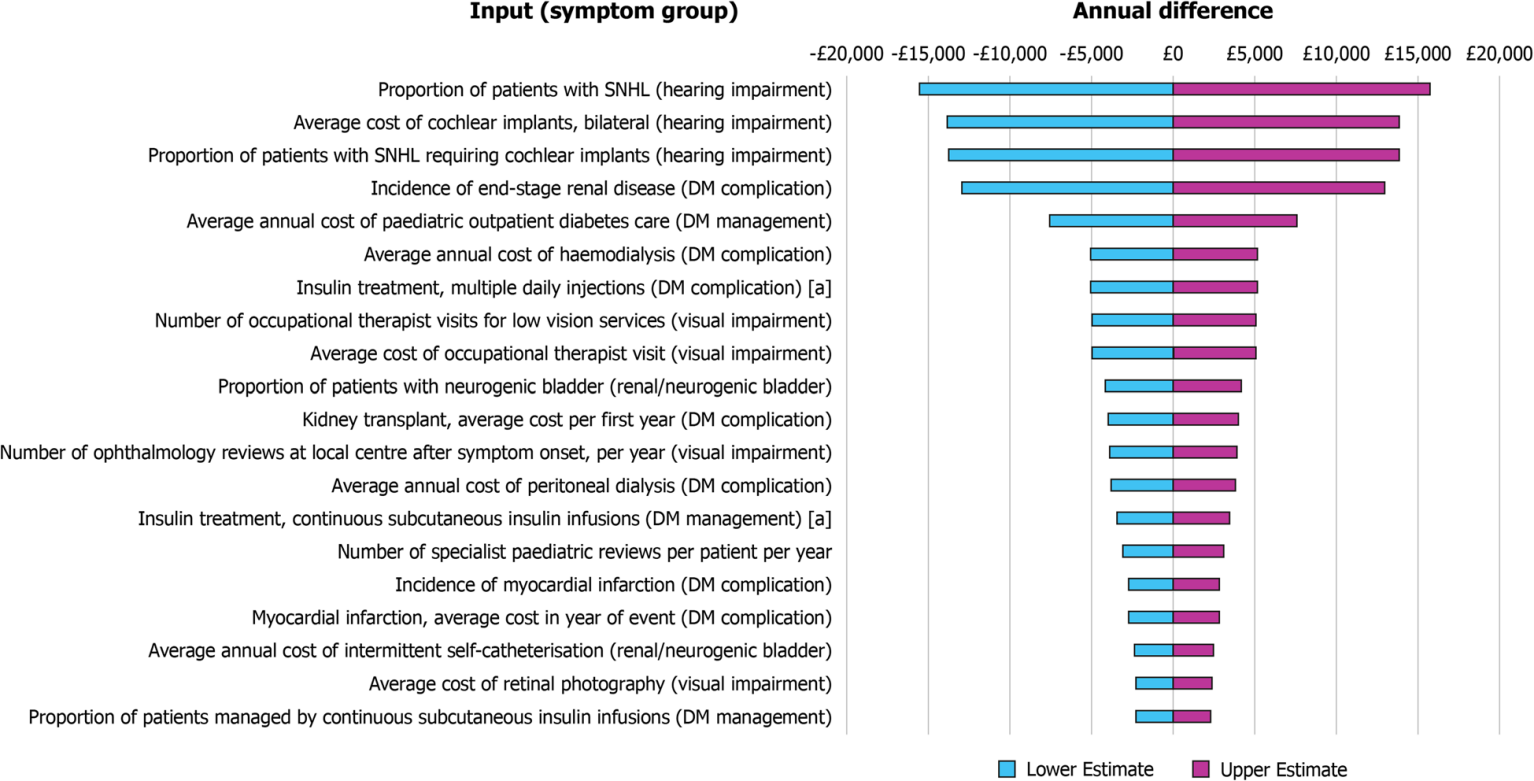
Tornado plot: the twenty greatest cost drivers identified by the DSA. [a] Average cost of consumables. Upper estimate demonstrates the impact on the final COI by increasing the variable by 10%. Lower estimate demonstrates the impact on the final COI by decreasing the variable by 10%. *COI* cost of illness, *DM* diabetes mellitus, *SNHL* sensorineural hearing loss

The DSA identified costs associated with hearing impairment and DM as major drivers in the model (Fig. 4). The results from the COI model were robust to uncertainty in the input parameters, as the total costs stayed within ±1.5% of the original point estimate, even when the most influential model inputs were varied by ±10%. These inputs are listed in Table 3.

**Table 3:** Values of the most influential COI model inputs

Input	Value	Reference
Population inputs		
Number of specialist paediatric reviews per patient per year	1	NHS Wolfram Syndrome Service (Birmingham Children’s Hospital), 2016 [16]
DM: management		
Paediatric outpatient diabetes care, average annual cost	£2925.00	NHS National Tariff 2016-2017 [20]
Proportion of patients managed by continuous subcutaneous insulin infusions	0.19	Diabetes UK, 2012 [7]
Insulin treatment, continuous subcutaneous insulin infusions, average annual cost of consumables	£2863.85	Cummins et al. 2010 [15]
Insulin treatment, multiple daily injections, average annual cost of consumables	£984.10	Cummins et al. 2010 [15]
DM: complications		
Incidence of end-stage renal disease	0.31	Pratoomsoot et la. 2009 [10]
Haemodialysis, average annual cost	£35,352.31	Roze et al. 2005 [26]
Kidney transplant, average cost per first year	£27,795.98	Roze et al. 2005 [26]
Peritoneal dialysis, average annual cost	£26,543.77	Roze et al. 2005 [26]
Incidence of myocardial infarction	0.32	Pratoomsoot et la. 2009 [10]
Myocardial infarction, average cost in year of event	£6234.64	Roze et al. 2005 [26]
Hearing impairment		
Proportion of patients with sensorineural hearing loss	0.66	Barrett et al. 1995 [2]
Proportion of patients with sensorineural hearing loss requiring cochlear implants	0.17	Karzon et al. 2013 [11]
Cochlear implants, bilateral, average cost (management)	£31,481.77	NHS Reference Costs 2015–2016 [19]
Visual impairment		
Number of ophthalmology reviews at local centre after symptom onset, per year	2.00	Patient experience (Wolfram Focus Group), 2016 [30]
Number of occupational therapist visits for low vision services	6.00	Warren 1995 [28]
Cost of occupational therapist visit	£131.02	NHS Reference Costs 2015–2016 [19]
Retinal photography, average cost	£174.16	NHS Reference Costs 2015–2016 [19]
Renal/neurogenic bladder		
Proportion of patients with neurogenic bladder	0.55	Barrett et al. 1995 [2]
Intermittent self-catheterisation, mean annual cost	£2771.58	NCGC Infection prevention and control, 2012 [12]

*DM* diabetes mellitus, *NCGC* National Clinical Guideline Centre, *NHS* National Health Service

## Discussion

Despite being a rare disease affecting fewer than 100 individuals in the UK, the current COI model found that Wolfram syndrome costs the NHS over £1 million annually. The model consolidated information on the disease and treatment pathway from a variety of sources, including care providers, medical experts and national statistics. The costs identified were across all areas of care, from diagnosis, to standard care and late-stage complications. This reflects the multisystemic and progressive nature of Wolfram syndrome, as well as the many various tests, screening and multidisciplinary interventions required by individuals throughout their lives [1]. The combination of this and the range of debilitating symptoms has a profound impact on health, quality of life and caregiver burden.

The annual cost per person with Wolfram syndrome estimated by the COI model (£16,498) is roughly comparable with COIs previously estimated for other ultra-rare diseases, although the COI varies widely between diseases [5, 31]. A COI study of Niemann-Pick disease (NPD), an ultrarare disease with a prevalence of 1:150,000 [32], estimated the average annual UK cost per person with NPD to be £18,012 in 2008 [33]. COI studies for other rare diseases have also been reported, including for cystic fibrosis (CF), where the annual direct treatment costs per person with CF reported ranged from €7108 to €51,551 in 2010, depending on country, age of patients and advances in standard of care [5, 31].

A key result of the Wolfram syndrome COI study is the identification of DM management as a major cost driver, which highlights the need for effective treatments which could potentially mitigate such consequences. The current lack of proven treatment options means that there is an imminent need for effective interventions to slow down or halt the progression of this life-shortening disease [2]. As with any rare disease, future directions for the treatment of Wolfram syndrome include investigations into drug repurposing, as well as novel drug development. One example is sodium valproate, which has shown promise for the treatment of Wolfram syndrome in experimental models [34] by mitigating the consequence of low levels of wolframin protein in people with Wolfram syndrome [35]. In cells that would ordinarily have high levels of wolframin, such as those in the pancreas or brain, the reduction in wolframin causes increased levels of cell death (apoptosis). Sodium valproate may reduce apoptosis in these wolframin-deficient models, in doing so slowing progression or ameliorating some symptoms of Wolfram syndrome [35]. Limiting disease progression would reduce the symptom-related costs and therefore reduce the economic burden, motivating further studies into sodium valproate as a potential Wolfram syndrome treatment.

Since the early 2000s, there has been a considerable increase in expenditure on research and development into treatments for rare diseases, likely aided by regulatory frameworks such as the Orphan Medicinal Products Regulation (2000) in the EU [36]. Despite this drive to develop new treatments for rare diseases, there is a lack of robust clinical, economic and epidemiological data for most rare diseases [31]. This limits the knowledge of the existing unmet need, and therefore the potential real-world impact of new treatments, including their likely effect on healthcare budgets if those treatments are introduced. Wolfram syndrome is no exception in terms of the lack of existing data in the literature. To our knowledge, this is the first COI model to be developed for this condition, providing a much-needed estimate of the healthcare burden of Wolfram syndrome in the UK, while also incorporating an expert-informed evaluation of the steps and resources involved in the care of individuals with this rare disease.

The lack of published COI data emphasises the need for studies such as this, but in turn means that there was a limit on the number of published sources from which to derive and verify inputs into the model. Expert opinion from the specialist centre at the NHS Wolfram Syndrome Service (Birmingham Children’s Hospital) was utilised to gain knowledge of factors not covered in the public domain to account for this lack of published data. By using this Wolfram syndrome expertise to inform the selection of model parameters, there can be greater confidence in the accuracy of estimates of the annual cost of Wolfram syndrome disease. However, with fewer than 10 new individuals with Wolfram syndrome diagnosed per year, the age distribution of affected individuals can vary year-on-year (Fig. 2), which will affect the symptoms and disease progression stages observed in clinical practice in any given year. Given this variability in age distribution, the use of the median age of symptom onset to estimate the number of individuals with a particular symptom could result in an underestimation of the number of complications in the Wolfram syndrome population. However, this is unlikely to have substantially impacted the model results, given that median ages of onset were not found to be within the top twenty cost drivers in the model (Fig. 4). All individuals in the model are assumed to have Wolfram syndrome type 1; individuals with Wolfram syndrome type 2 would slightly differ symptomatically, however, these individuals are not typically seen in the UK setting and thus this assumption is unlikely to bias the results. It is also possible that some of the patients diagnosed with Wolfram syndrome may have been misdiagnosed and therefore should not have been considered by this study.

The model considers a healthcare payer perspective, and therefore only represents the direct costs of Wolfram syndrome to the NHS. This is a clear limitation of the study as the substantial burden of the disease on affected families, and the resulting costs to them as well as wider society, were not included in this model (for example, costs associated with provision of teaching support for the visually impaired in schools or attending colleges for the blind). However, inclusion of indirect costs was deemed impractical for this study due to a lack of available information to determine appropriate study inputs. Establishing an effective treatment regimen for each individual can be a slow and time-consuming process. Costs associated with lost time, such as lost earnings, were not included in the model. A focus group of individuals with Wolfram syndrome highlighted that a major financial cost to families is loss of working hours, often due to extensive time off for illness, appointments and caring responsibilities [30]. Furthermore, frequent travel, in addition to accommodation may incur significant out-of-pocket expenditure for parents and carers. The existence of centralised services such as the Birmingham centre is valuable to both researchers and patients alike, as specialist centres are likely to offer a setting for rare disease research as well as to help standardise the treatment strategies and improve the overall standard of healthcare provided to those with the disorder.

It is important to highlight that the limitations noted above are common to all COI models that only present a healthcare payer perspective. As the first COI model developed for Wolfram syndrome, it represents a crucial step forward in understanding the true cost of this disease to the NHS. The model accurately reflects the diagnosis process and symptom progression followed by these patients, as validated by clinical experts. The model provides a detailed view of the cost of this illness and offers a novel tool to aid in identifying potential areas of cost savings.

A number of potential policy implications come with the publication of this Wolfram syndrome COI data. Both the UK Strategy for Rare Diseases [37] and the EU-supported RARE-Bestpractices program [38] have indicated the value in undertaking research to address the gaps in knowledge and to help to define the best care pathways for rare diseases. Results from health economic studies such as this one can inform evidence-based policies, and as a result, help to ensure that patients across the UK and beyond receive the same, high quality standard of care.

## Conclusions

This study is the first COI model for Wolfram syndrome and provides important information to facilitate economic evaluation of prospective therapies for this disease. The costs associated with DM care and late-stage complications of DM, hearing impairment and visual impairment made the greatest contribution to the final COI. These findings add much-needed information to a scarce evidence base, although additional research into the indirect costs associated with this disease is recommended.

## Data Availability

The datasets supporting the conclusions of this article are included within the article.

## Abbreviations

ABR: Auditory brain response
BNF: British National Formulary
CCG: Clinical Commissioning Group
CF: Cystic fibrosis
*CISD2*: CDGSH iron sulphur domain protein 2 (gene)
COI: Cost of illness
D: Deafness
DI: Diabetes insipidus
DIDMOAD: Diabetes insipidus, diabetes mellitus, optic atrophy and deafness (alternative name for Wolfram syndrome)
DM: Diabetes mellitus
DSA: Deterministic sensitivity analysis
EMA: Endomysial antibodies
ENT: Ear, nose and throat
GP: General practitioner
HbA1c: Haemoglobin A1c
IgA: Immunoglobulin A
MDT: Multi-disciplinary team
MI: Myocardial infarction
MRI: Magnetic resonance imaging
NCGC: National Clinical Guideline Centre
NHS: National Health Service
NPD: Niemann-Pick disease
OA: Optic atrophy
OT: Occupational therapist
PSSRU: Personal Social Services Research Unit
PY: Per year
SALT: Speech and Language Therapists
SC: Subcutaneous
SNHL: Sensorineural hearing loss
SPH: Sphere
tTG: Tissue transglutaminase
UTI: Urinary tract infection
*WFS1*: Wolfram syndrome type 1 (gene)

## References

[1] Orphanet. The portal for rare diseases and orphan drugs, Wolfram syndrome. Available from: http://www.orpha.net/consor/cgi-bin/OC_Exp.php?lng=EN&Expert=3463. Last accessed 19 Oct 2018.

[2] Barrett TG, Bundey SE, Macleod AF (1995). Neurodegeneration and diabetes: UK nationwide study of Wolfram (DIDMOAD) syndrome. Lancet..

[3] Chaussenot A, Bannwarth S, Rouzier C, Vialettes B, Mkadem SA, Chabrol B, Cano A, Labauge P, Paquis-Flucklinger V (2011). Neurologic features and genotype-phenotype correlation in Wolfram syndrome. Ann Neurol.

[4] Urano F (2016). Wolfram syndrome: diagnosis, management, and treatment. Curr Diab Rep.

[5] Barrett TG, Bundey SE (1997). Wolfram (DIDMOAD) syndrome. J Med Genet.

[6] Kinsley BT, Swift M, Dumont RH, Swift RG (1995). Morbidity and mortality in the Wolfram syndrome. Diabetes Care.

[7] National Health Service. Diabetes UK. Available from: https://jdrf.org.uk/wp-content/uploads/2015/10/The_United_Kingdom_Insulin_Pump_Audit_May_2013.pdf. Last accessed 19 Oct 2018.

[8] Rohayem J, Ehlers C, Wiedemann B, Holl R, Oexle K, Kordonouri O, Salzano G, Meissner T, Burger W, Schober E, Huebner A, Lee-Kirsch MA (2011). Diabetes and neurodegeneration in Wolfram syndrome: a multicenter study of phenotype and genotype. Diabetes Care.

[9] Tranebjaerg L (2009). WFS1-related disorders.

[10] Pratoomsoot C, Smith HT, Kalsekar A, Boye KS, Arellano J, Valentine WJ (2009). An estimation of the long-term clinical and economic benefits of insulin lispro in type 1 diabetes in the UK. Diabet Med.

[11] Karzon RK, Hullar TE (2013). Audiologic and vestibular findings in Wolfram syndrome. Ear Hear.

[12] National Health Service. NCGC 2012 (2010). Available from: https://www.nice.org.uk/guidance/cg148/evidence/full-guideline-188123437. Last accessed 19 Oct 2018.

[13] Swift RG, Sadler DB, Swift M (1990). Psychiatric findings in Wolfram syndrome homozygotes. Lancet..

[14] British National Formulary. Available from: https://www.bnf.org/products/bnf-online/. Last accessed 19 Oct 2018.

[15] Cummins E, Royle P, Snaith A, Greene A, Robertson L, McIntyre L, Waugh N (2010). Clinical effectiveness and cost-effectiveness of continuous subcutaneous insulin infusion for diabetes: systematic review and economic evaluation. Health Technol Assess.

[16] Expert Opinion (2016). NHS Wolfram Syndrome Service (Birmingham Children’s Hospital).

[17] Expert Opinion (2016). NHS Wolfram Syndrome Service (Queen Elizabeth Hospital).

[18] Federation of (Ophthalmic and Dispensing) Opticians. Available from: http://www.fodo.com. Last accessed 19 Oct 2018.

[19] National Health Service. Reference costs (2015–2016). Available from: https://www.gov.uk/government/publications/nhs-reference-costs-2015-to-2016. Last accessed 19 Oct 2018.

[20] National Health Service. National Tariff (2016-2017). Available from: https://www.gov.uk/government/publications/nhs-national-tariff-payment-system-201617. Last accessed 19 Oct 2018.

[21] National Health Service. Vale of York CCG. Available from: https://www.valeofyorkccg.nhs.uk/. Last accessed 19 Oct 2018.

[22] National Health Service. Drug Tariff (May 2016). Available from: https://www.nhsbsa.nhs.uk/pharmacies-gp-practices-and-appliance-contractors/drug-tariff. Last accessed 19 Oct 2018.

[23] National Health Service. Bolton Foundation Trust (2015). Available from: http://www.boltonft.nhs.uk. Last accessed 19 Oct 2018.

[24] National Health Service. Southern Derbyshire CCG 2016. Available from: https://www.derbyhospitals.nhs.uk. Last accessed 19 Oct 2018.

[25] Personal Social Services Research Unit. Available from: http://www.pssru.ac.uk/project-pages/unit-costs/2016/. Last accessed 19 Oct 2018.

[26] Roze S, Valentine WJ, Zakrzewska KE, Palmer AJ (2005). Health-economic comparison of continuous subcutaneous insulin infusion with multiple daily injection for the treatment of type 1 diabetes in the UK. Diabet Med.

[27] UK Genetic Testing Network. Available from: https://ukgtn.nhs.uk. Last accessed 19 Oct 2018.

[28] Warren M (1995). Providing low vision rehabilitation services with occupational therapy and ophthalmology: a program description. Am J Occup Ther.

[29] Wolfram Syndrome Guideline Development Group. Management of Wolfram Syndrome A Clinical Guideline. 2014. Available from: http://www.orpha.net/national/data/IE-EN/www/uploads/Wolfram2014.pdf. Last accessed 04 July 2019.

[30] Findacure. Patient focus group report: Wolfram syndrome. 2016. Available from: https://www.findacure.org.uk/wp-content/uploads/2018/06/Wolfram-syndrome-Findacure-Rare-Disease-Perspectives-2016.pdf. Last accessed 04 July 2019.

[31] Angelis A, Tordrup D, Kanavos P (2015). Socio-economic burden of rare diseases: a systematic review of cost of illness evidence. Health Policy.

[32] Patterson M (1993). Niemann-pick disease type C. GeneReviews®.

[33] Imrie J, Galani C, Gairy K, Lock K, Hunsche E (2009). Cost of illness associated with Niemann-pick disease type C in the UK. J Med Econ.

[34] Kakiuchi C, Ishigaki S, Oslowski CM, Fonseca SG, Kato T, Urano F (2009). Valproate, a mood stabilizer, induces WFS1 expression and modulates its interaction with ER stress protein GRP94. PLoS One.

[35] Terasmaa A, Soomets U, Oflijan J, Punapart M, Hansen M, Matto V, Ehrlich K, Must A, Koks S, Vasar E (2011). Wfs1 mutation makes mice sensitive to insulin-like effect of acute valproic acid and resistant to streptozocin. J Physiol Biochem.

[36] European Parliament and of the Council Regulation (EC) 141/2000 of 16 December 1999 on Orphan Medicinal Products. OJL 018/1, 22.01.2000. Available from: https://eur-lex.europa.eu/LexUriServ/LexUriServ.do?uri=OJ:L:2000:018:0001:0005:en:PDF. Last accessed 19 Oct 2018.

[37] UK Department of Health. The UK Strategy for Rare Diseases. Available from: http://www.raredisease.org.uk/uk-strategy-for-rare-diseases/. Last accessed 19 Oct 2018.

[38] RARE-Bestpractices. Available from: http://www.rarebestpractices.eu/. Last accessed 19 Oct 2018.

